# Prevalence of HIV, hepatitis B, and hepatitis C in people with severe mental illness: a systematic review and meta-analysis

**DOI:** 10.1016/S2215-0366(15)00357-0

**Published:** 2016-01

**Authors:** Elizabeth Hughes, Shaan Bassi, Simon Gilbody, Martin Bland, Fabiola Martin

**Affiliations:** a University of Huddersfield, Huddersfield, UK; b South West Yorkshire Partnership NHS Foundation Trust, Wakefield, UK; c University of York, York, UK; d Blueprint Partnership, Manchester, UK

## Abstract

**Background:**

Although people with serious mental illnesses have a high risk of contracting blood-borne viral infections, sexual health has largely been neglected by researchers and policy makers involved in mental health. Failure to address this shortcoming could increase morbidity and mortality as a result of undetected and untreated infection. We did a systematic review and meta-analysis to estimate the prevalence of blood-borne viral infection in people with serious mental illness.

**Method:**

We searched the Cochrane Library, Medline, Embase, PsycInfo, CINAHL, and DARE for studies of the prevalence of HIV, hepatitis B virus, and hepatitis C virus in people with serious mental illness, published between Jan 1, 1980, and Jan 1, 2015. We group prevalence data by region and by virus and estimated pooled prevalence. We did a sensitivity analysis of the effect of study quality on prevalence.

**Findings:**

After removal of duplicates, we found 373 abstracts, 91 of which met our eligibility criteria. The prevalences of blood-borne viral infections in people with serious mental illness were higher than in the general population in places with low prevalence of blood-borne viruses, such as the USA and Europe, and on par with the general population in regions with high prevalence of blood-borne viruses (Africa for HIV and southeast Asia for hepatitis B virus and hepatitis C virus). Pooled prevalence of HIV in people with serious mental illness in the USA was 6·0% (95% CI 4·3–8·3). Sensitivity analysis showed that quality scores did not significantly affect prevalence.

**Interpretation:**

People with serious mental illness are at risk of blood-borne viral infections. However, because of methodological limitations of the studies the prevalence might be overestimated. Serious mental illness is unlikely to be a sole risk factor and risk of blood-borne viral infection is probably multifactorial and associated with low socioeconomic status, drug and alcohol misuse, ethnic origin, and sex. Health providers should routinely discuss sexual health and risks for blood-borne viruses (including risks related to drug misuse) with people who have serious mental illness, as well as offering testing and treatment for those at risk.

**Funding:**

Wellcome Trust.

## Introduction

240 million people have a serious mental illness, with a broadly similar distribution worldwide.^1^ Serious mental illness is defined as a diagnosis of mental illness (eg, schizophrenia and schizoaffective disorders, bipolar disorder, or psychosis) that is persistent, disabling, and requiring specialised psychiatric treatment as an outpatient or inpatient admission.^1^ The point prevalence of serious mental illness is 4·6 cases per 1000 people, and 4·0% of people have a serious mental illness at some point during their life.^1^

With the increasing evidence that people with serious mental illness have significant health inequalities,^2^ increasing prominence has been given to physical health screening, health education, and improving access to treatment in primary and secondary care. However, sexual health needs, including screening for and prevention of sexually transmitted infections and blood-borne viruses, are neglected in this population. Of particular concern is the higher risk of blood-borne virus infections (HIV, hepatitis B virus, and hepatitis C virus), shown by prevalence studies done over the past 30 years.^3^, ^4^ These viruses are transmitted by parenteral contact with contaminated body fluids (blood and blood products; contaminated instruments and needles; semen and vaginal fluids). Transmission can also occur through unprotected sex (anal, vaginal, or oral), vertical transmission from mother to baby, and by sharing drug injecting equipment.

HIV, hepatitis B, and hepatitis C are serious infections, but can be treated. The prognosis is much improved by earlier detection and treatment. Prevalence studies^3^, ^5^, ^6^ have shown that serious mental illness is a risk factor for blood-borne virus infection. Many people with serious mental health problems engage in behaviours that increase their risk of infection with blood-borne viruses, including unprotected sex with multiple partners, sex work (or sex trading—performing sexual acts in exchange for a commodity), and intravenous drug use (or having a sexual partner who is an injecting drug user). Further risk can result from hypersexuality during an acute phase of mental illness, as well as co-occurring substance misuse problems that can lead to sexual risks while intoxicated. Finally, people with serious mental illness who live in shared accommodation might share personal equipment (eg, razors, toothbrushes), which might increase the risk of hepatitis B and hepatitis C transmission.

In the UK, 93 000 men and 40 000 women are infected with HIV, and in 2013, there were 6000 new cases of HIV.^7^ In 2012, the incidence of hepatitis B in England was 1·4 cases per 100 000 people per year and prevalence was 0·1–0·5%.^8^ In 2009, the overall incidence of reported acute hepatitis B in the USA^9^ was 1·5 per 100 000 people per year and 800 000–1·4 million people in the USA have chronic hepatitis B virus infection. 786 000 people worldwide die from hepatitis B virus-related liver disease each year.^10^, ^11^ About 3% of the world's population are infected with hepatitis C virus, with about 4 million carriers in Europe alone^12^ and 214 000 in the UK.^10^ There were 17 000 new cases of hepatitis C in the USA in 2007, with 3·2 million people infected in total.

Although previous reviews of blood-borne infection in people with serious mental illness have been published,^3^, ^5^, ^6^ no systematic reviews have been done and the reviews rarely reported the rate of HIV, hepatitis B, and hepatitis C.^13^ We did a systematic review and meta-analysis of prevalence studies to understand the global prevalence of HIV, hepatitis B virus, and hepatitis C virus in people with serious mental illness.

## Methods

### Search strategy

We searched the Cochrane Library, Medline, Embase, PsycInfo, CINAHL, and DARE for studies published in English between Jan 1, 1980, and June 5, 2012, with the terms “hepatitis C”, ‘HCV’, “hepatitis B”, ‘HBV’, “HIV”, “human immunodeficiency virus”, “blood borne virus” cross-referenced with “bipolar”, “psychiatr*”, “schizophreni*”, “psychosis”, “schizoaffective”, “mental patient*”, “mental illness”, and “mental disorder*”. We also included eligible studies cited in reports identified by our database search. We repeated the search for June 5, 2012, to Jan 1, 2015, and identified two more papers.

### Data collection

We systematically searched the scientific literature for observational cross-sectional studies that reported the seroprevalence of HIV, hepatitis B virus, or hepatitis C virus according to opt-in, opt-out, or anonymous unlinked blood or other bodily fluids research methods, in people aged older than 15 years, diagnosed with serious mental illness, and treated in a psychiatric setting. We excluded studies in which prevalence data were only obtained from case notes or only from self-report (not independently verified by testing). We did not include grey literature.

After removing duplicates, SB screened the titles and abstracts using the eligibility criteria, with independent verification by EH and FM. For studies deemed suitable, we obtained the full text and they were again scrutinised against the eligibility criteria by SB and verified independently by EH and FM. Reports about which there was uncertainty were discussed by FM and EH with SB until a consensus about eligibility was achieved. We extracted data from eligible full-text articles including study characteristics, study date, publication date, location, diagnostic criteria, demographics (age, sex, ethnicity), consent, consent rate, ethics approval, post-test treatment, sample size, testing procedure, and prevalence.

We used the Quality Assessment Tool for Systematic Reviews of Observational Studies to assess the quality of the data.^14^ This instrument is reliable compared with other quality assessment tools.^14^ We modified the tool and each report was scored as follows: whether participants were clearly defined as representing the serious mental illness population (yes=1, no=0); participation rate (response rate >60%=1, response rate ≤60% or not reported=0); whether investigators controlled for confounding (eg, stratification, matching, restriction, adjustment) when analysing associations (controlled=1, only descriptive=0); and sample size (≥200 participants=1, <200 participants=0).

### Data analysis

We did a meta-analysis to calculate combined estimates and 95% CIs for each continent separately. We did logistic regression to allow for the difficulties caused by proportions being unable to have values less than 0. We assumed random effects because there was clear clinical heterogeneity between the populations sampled. We did the calculations using Comprehensive Meta-Analysis 2. We transformed logits of estimated prevalence and their 95% CIs back to percentages. We prepared forest plots using Stata (version 12). We calculated relative weights for each continent, so that the weights for each continent sum to 100.

We did a sensitivity analysis relating to quality scores using Stata 13. The outcome variable was the logit-transformation of prevalence. We did two such analyses: one for all studies with quality score as a quantitative predictor, and one for all studies using quality score as a quantitative predictor and region as a qualitative predictor. The results are presented as odds ratio (OR) per unit increase in quality score with 95% CIs.

## Results

**Figure 1:**
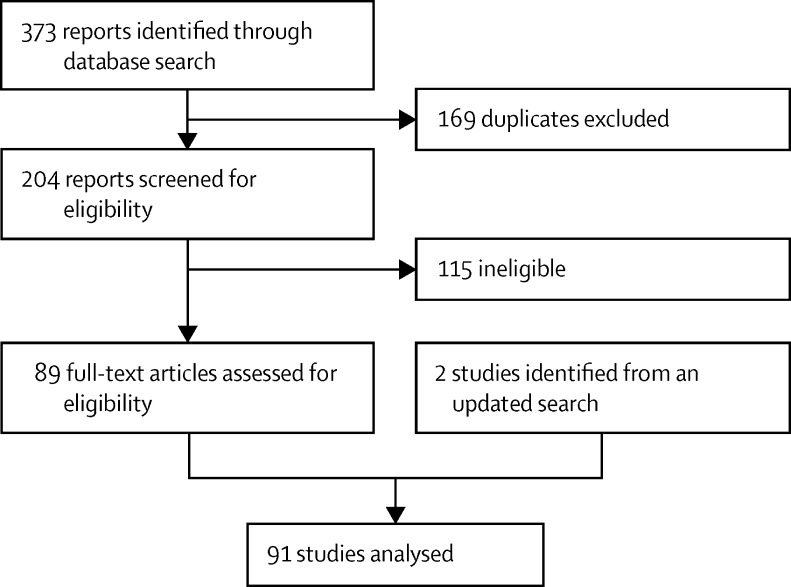
Study selection

Our literature search identified 373 reports, 169 of which were duplicates (figure 1). 41 publications were excluded because the full text was not available in English, followed by another 74 that were ineligible. With the addition of two papers from an updated search, we had 91 articles for quality assessment and meta-analysis.

**Figure 2:**
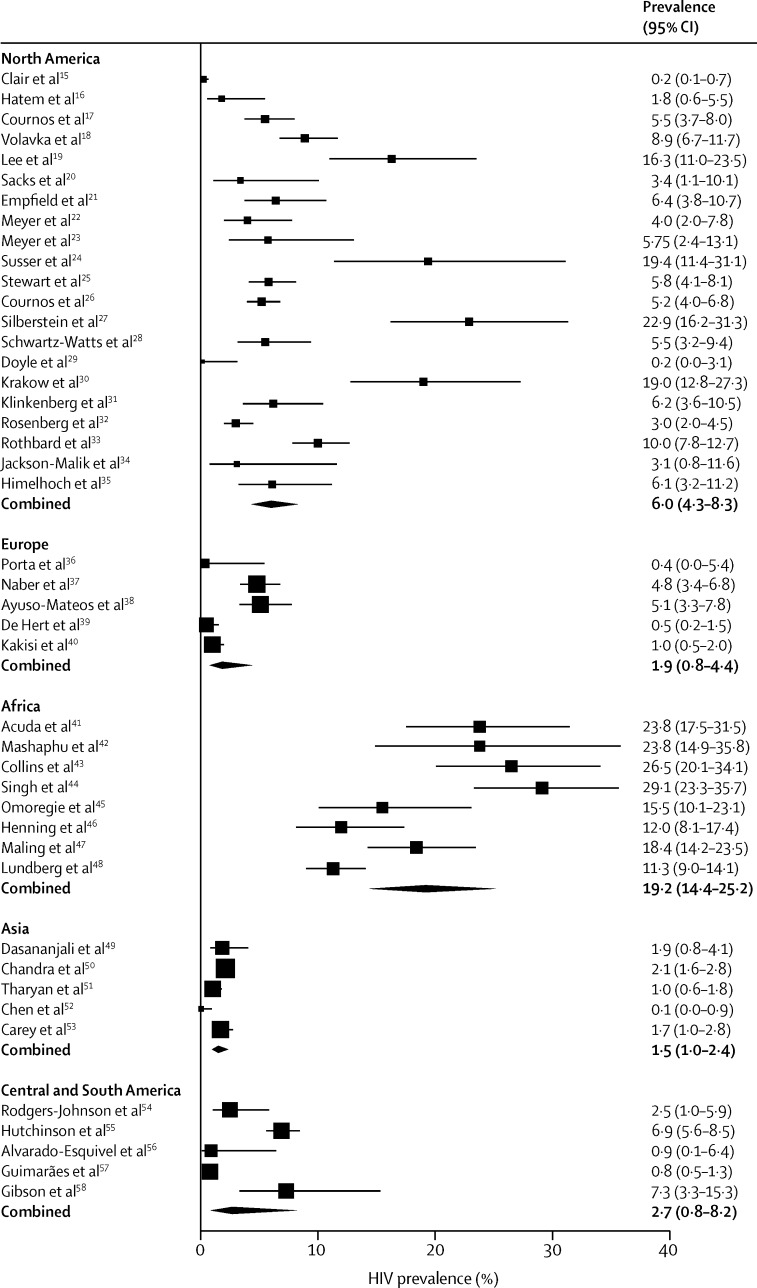
Prevalence of HIV in people with serious mental illness

44 studies assessed HIV infection (table 1), including a total of 21 071 patients. The pooled prevalence of HIV was highest in Africa (19%, 95% CI 14–25) and it was 2% in Europe and 6% in the USA (Table 1, Table 2, figure 2). Few data were available from Europe, Central and South America, and Asia, and the prevalence of HIV was very poorly recorded in these regions. Only three studies were done in India,^51^, ^53^ and only one study,^38^ done more than 20 years ago, was from Spain.

**Figure 3:**
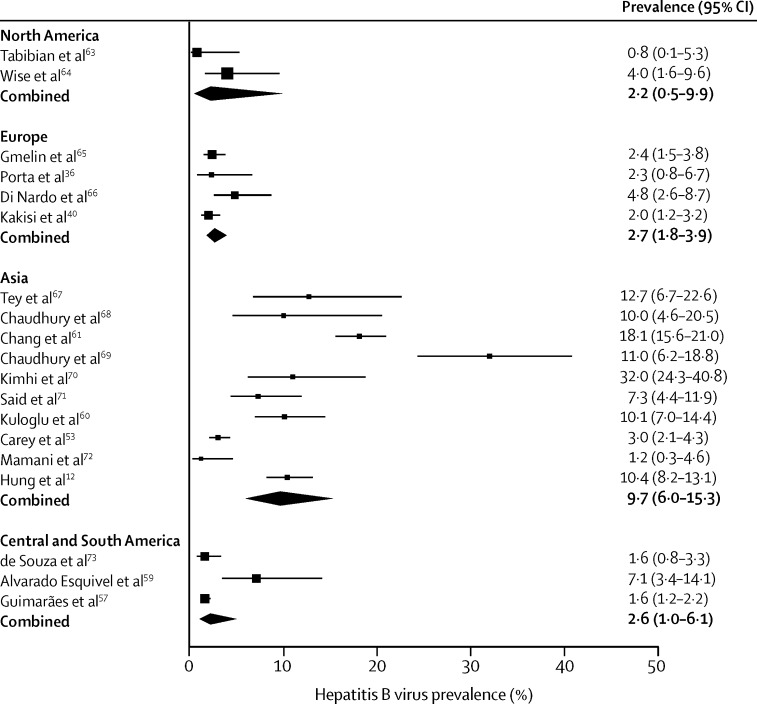
Prevalence of hepatitis B virus in people with serious mental illness

19 studies reported prevalence of hepatitis B virus,^53^, ^57^, ^59^, ^60^, ^61^, ^62^ including a total of 8163 patients with serious mental illness tested for hepatitis B virus (table 3). The pooled prevalence of hepatitis B virus was 2·2% (95% CI 0·5–9·9) in North America, and 9·7% (95% CI 0·6–15·3) in Asia (table 2, figure 3). A study from Turkey^60^ reported 51% hepatitis B virus prevalence with 10% HBsAg positivity indicating active infection; the virus is highly prevalent in the general population of Turkey. A study from Taiwan^61^ reported an 18% prevalence of HBsAg, which is consistent with the general population: hepatitis B virus infection is endemic in Taiwan, with 80–90% of adults infected.

**Figure 4:**
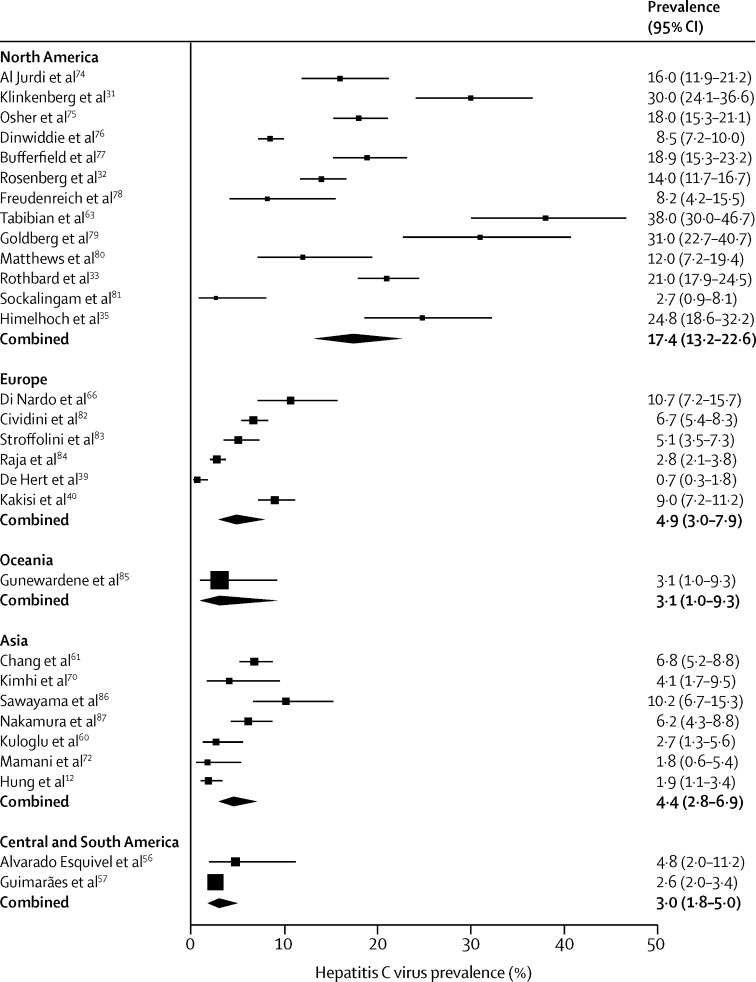
Prevalence of hepatitis C virus in people with serious mental illness

28 studies tested 14 888 patients with serious mental illness for hepatitis C virus (Table 2, Table 4, figure 4). The prevalence of hepatitis C in people with serious mental illness was greatest in Turkey, perhaps a result of the high prevalence in the general population in Turkey. Pooled data from 13 studies from North America gave a prevalence of 17·4% (95% CI 13·2–22·6), which is higher than in the general population, of whom roughly 1% are infected (2·7 million).^88^ In Asia, pooled hepatitis C virus prevalence was 4·4% (95% CI 2·8–6·9). However, these data are from large and diverse geographical areas including southeast Asia and Turkey.

Most studies consisted of convenience samples of people recruited from a particular treatment setting, typically inpatient psychiatric care. Although all the studies included patients with serious mental illness, the proportions of specific diagnoses in each sample varied. We assessed the effect of study quality on virus prevalence by meta-regression on quality score, for all studies combined and adjusting for geographical region. No analysis showed a significant effect of study quality on prevalence (table 5).

Most of the studies had additional data on risk factors for blood-borne viruses, such as intravenous drug use and sexual behaviour, to test associations with infection. The reporting and the nature of these risk factors varied widely. Infomation on risk factors was mainly extracted from routine clinical case notes as opposed to using a standardised risk tool.^89^ Because of the variability of data quality and reporting consistency, we could not calculate adjusted prevalence after controlling for these risk factors. However, three common factors seem to increase the likelihood of infection with a blood-borne virus: first, being black and female;^17^, ^26^ second, injecting drug use;^32^ and third, engaging in risky sexual behaviour, including not using a condom, having multiple partners, sex trading, and unprotected sex with a partner who is infected with a blood-borne virus.

## Discussion

Our aim was to estimate the prevalence of blood-borne infection in people with serious mental illness. Most of the studies were of moderate to low quality, and based on convenience samples drawn from treatment settings rather than representative samples. This sampling method means that the prevalence reported was possibly overestimated. However, a study in Brazil, which used a representative sample drawn from the community as well as treatment settings, still showed that blood-borne infections are common in people with serious mental illness.^57^ The quality of defining the sample in terms of diagnoses of mental illness varied. Many studies used case note diagnoses rather than independently verified diagnoses made with gold standard diagnostic tools. Inpatient settings are likely to treat the most acutely ill people often with complex needs and a history of substance misuse.

The prevalences of blood-borne viruses in people with serious mental illness were consistently higher than in the general population in regions with a low prevalence of blood-borne viruses, such as North America and Europe, and on par with the general population in regions with high general prevalence such as Africa for HIV and southeast Asia for hepatitis B virus and hepatitis C virus. The estimated prevalence of HIV in people with serious mental illness in the USA was 6% (95% CI 4·3–8·3), which is considerably higher than the 0·6% of the general population of the USA who have HIV.^90^

However, serious mental illness might not be an isolated risk factor for blood-borne virus infection, but might be better thought of as a potentially confounded association with poor socioeconomic background, drug and alcohol misuse, sex, and ethnic origin.

Three USA studies^18^, ^21^, ^26^ included odds ratios adjusted for risk factors and showed that they significantly increased the risk of HIV and other blood-borne viral infections. However, these studies were done in settings where dual diagnosis of substance misuse and mental illness is very common. The samples were drawn from psychiatric inpatient and outpatient services in deprived urban areas with substantial social deprivation and health inequality, especially in those of non-white ethnic backgrounds.

Several studies, from both high prevalence and low prevalence locations, individually found a positive association between sex and infection. Women had a significantly higher risk of HIV infection than did men drawn from the same populations. One explanation might be that women with serious mental illness are more likely to experience exploitation and sexual assault, as well as power differentials, making them less empowered to negotiate condom use or to refuse sex.^91^ By contrast, men with serious mental illness were more likely to carry hepatitis B virus or hepatitis C virus, which could be because injecting drug use is more common in men. However, the causes of these sex differences were probably multifactorial, which we could not assess because of the heterogeneity of geography, demographics, and risk factors in the studies we included.

Many of the studies have been done in the USA, with fewer located in other countries. Of particular note is the paucity of research in Europe, and there have been no prevalence studies done in the UK. However, two articles suggest a potential problem in the UK. A hepatitis C virus screening and referral project done by an assertive outreach mental health team^92^ showed more than expected infections amongst users of the service. Of 76 users, ten (13%) were hepatitis C virus positive, and almost half had a history of intravenous drug use. Another article^93^ reported on the acceptability and feasibility of offering testing for blood-borne viruses in psychiatric inpatient settings. The results suggest more HIV, hepatitis B virus, and hepatitis C virus in patients who participated in the study. Overall, 18% of participants had current or past exposure to a blood-borne virus, one of whom was newly diagnosed with HIV and three were newly diagnosed with hepatitis B virus. Therefore, there is an urgent need to undertake high quality epidemiological studies of blood-borne virus infections and their associated risk behaviours in the people with serious mental illness in the UK and northern Europe.

Few studies systematically collected data for risk factors directly from the participants. The risk data were mainly collected from case notes and routine clinical record systems. Sexual and drug use behaviours are probably under-reported in case notes, because there is evidence that mental health services do not consistently assess these behaviours in routine care.^94^, ^95^ Without accurate and consistent measurement of risks, we could not calculate the effect of the risk factors as mediators of infection in this population, and have merely mentioned the factors identified by individual studies that warrant more rigorous investigation. There is a need for a prospective longitudinal study of a cohort of people with serious mental illness, which can track risk behaviour and infections powered sufficiently to identify the mediating factors between serious mental illness and blood-borne virus infection.

We included cross-sectional studies. None of the studies included a matched comparison group of people without serious mental illness. Prospective cohort studies are needed that use representative samples alongside matched controls of people without serious mental illness. Such studies are the only way to accurately test whether the prevalence of blood-borne viruses is significantly elevated in people with serious mental illness compared with the general population. Comparing the estimated prevalence with available data for the country or region is limited but it does offer some indication that prevalence is higher in people with serious mental illness. The prevalence of HIV infection in the general population is much lower in the UK^96^ than in the USA, and therefore the assumption is that HIV infection is less of a risk for people with serious mental illness who live in the UK. However, hepatitis C virus is prevalent in drug users in the UK,^97^ and there could be a risk of hepatitis C virus infection and co-infections in people with serious mental illness as a result of substance misuse.^98^

This meta-analysis estimated pooled prevalence of HIV, hepatitis B virus, and hepatitis C virus in people with serious mental illness. Our review included only published work, and therefore might have missed studies yet to be reported. In addition, the search strategy included only reports published in English, which might have biased our findings towards English-speaking countries.

It is unclear why sexual health has been neglected as part of the physical health agenda for people with serious mental illness. One reason might be the perception that people with serious mental illness do not engage in activities that place them at risk, such as intravenous drug use or unprotected sex. However, 30–50% of people with serious mental illness have substance misuse disorders,^98^, ^99^ and, although intravenous drug use is rare, patients might have sexual partners who inject drugs, facilitating viral transmission. Additionally, as with the population as a whole, a substantial proportion of people with serious mental illness are sexually active and see intimate relationships as an important part of their lives.^5^, ^100^ The lack of attention of policy makers and educators has led to a lack of awareness and a failure to provide people with serious mental illness with access to assessment, screening, and education for sexually transmitted infections, including blood-borne viruses.

A qualitative study in London^101^ documented that most people with psychosis were engaged in seeking and forming intimate relationships. Additionally, some had negative and harmful relationship experiences, including sexual exploitation and violence, yet these issues were rarely part of their routine consultation with their health-care providers. This lack of attention to sexual health and safety has also been reported in a review,^91^ which found that although women with serious mental illness were twice as likely to be exposed to severe domestic violence compared with women in the general population, these incidents were rarely detected by the health-care services they attended. A survey of psychiatrists in a Sydney, Australia, mental health service^102^ found poor knowledge of hepatitis C virus, and clinicians perceived their patients to be at lower risk than prevalence studies suggest.^3^ A survey of mental health staff at a London NHS service^94^ also showed that workers underestimated the risk of HIV in people with schizophrenia. Although they reported feeling comfortable discussing sexual health, this rarely happened in practice.^94^ In addition, a qualitative study of Australian mental health nurses^95^ showed that discussions of sex and sexuality were generally avoided.

In summary, we show the high prevalence of blood-borne infections in people with serious mental illness, but more importantly we document the paucity of data on this topic. Although the physical health inequalities of people with serious mental illness have been identified and health policy is developing to ensure that these inequalities are addressed, little attention has been given to the sexual health and specifically risk factors facilitating transmission of blood-borne viruses in people with serious mental illness in the UK and worldwide. There is an urgent need for further robust epidemiological research using representative samples of people with serious mental illness to assess the relationship between lifestyle behaviour and risk of infections to more fully understand the relationship between serious mental illness and viral infection, and to inform preventive strategies in this population.

**This online publication has been corrected. The corrected version first appeared at thelancet.com/psychiatry on January 6, 2016**

## Figures and Tables

**Table 1:** Included studies of HIV in people with serious mental illness

	**Date**	**Location**	**Number of participants**	**Prevalence of HIV (%)**	**Quality score**
**North America**					
Clair et al^15^	1989	USA	1496	0·24	1
Hatem et al^16^	1990	USA	163	1·8	1
Cournos et al^17^	1991	USA	451	5·5	3
Volavka et al^18^	1991	USA	515	8·9	3
Lee et al^19^	1992	USA	135	16·3	0
Sacks et al^20^	1992	USA	87	3·4	1
Empfield et al^21^	1993	USA	203	6·4	2
Meyer et al^22^	1993	USA	199	4	2
Meyer et al^23^	1993	USA	87	5·75	1
Susser et al^24^	1993	USA	62	19·4	1
Stewart et al^25^	1994	USA	533	5·8	3
Cournos et al^26^	1994	USA	971	5·2	3
Silberstein et al^27^	1994	USA	118	22·9	2
Schwartz-Watts et al^28^	1995	USA	220	5·50	3
Doyle et al^29^	1997	USA	246	0	1
Krakow et al^30^	1998	USA	113	19	0
Klinkenberg et al^31^	2003	USA	204	6·2	2
Rosenberg et al^32^	2005	USA	755	3	3
Rothbard et al^33^	2009	USA	588	10	2
Jackson-Malik et al^34^	2011	USA	64	3·1	1
Himelhoch et al^35^	2011	USA	153	6·1	2
**Europe**					
Porta et al^36^	1990	Spain	139	0	0
Naber et al^37^	1994	Germany	623	4·8	1
Ayuso-Mateos et al^38^	1997	Spain	390	5·1	3
De Hert et al^39^	2009	Belgium	595	0·5	2
Kakisi et al^40^	2009	Greece	805	1	1
**Africa**					
Acuda et al^41^	1996	Zimbabwe	143	23·8	2
Mashaphu et al^42^	2007	South Africa	63	23·8	1
Collins et al^43^	2009	South Africa	151	26·5	3
Singh et al^44^	2009	South Africa	206	29·1	3
Omoregie et al^45^	2009	Nigeria	121	15·5	1
Henning et al^46^	2011	South Africa	195	12	2
Maling et al^47^	2011	Uganda	272	18·4	4
Lundberg et al^48^	2013	Uganda	602	11·3	4
**Asia**					
Dasananjali^49^	1994	Thailand	325	1·85	2
Chandra et al^50^	2003	India	2283	2·11	1
Tharyan et al^51^	2003	India	1160	1·03	3
Chen^52^	1994	Taiwan	834	0	2
Carey et al^53^	2007	India	948	1·7	4
**Central and South America**					
Rodgers-Johnson et al^54^	1996	Jamaica	201	2·5	2
Hutchinson et al^55^	1999	Trinidad and Tobago	1227	6·9	1
Alvarado-Esquivel et al^56^	2008	Mexico	105	0·9	0
Guimarães et al^57^	2009	Brazil	2238	0·8	3
Gibson et al^58^	2010	West Indies	82	7·3	1

**Table 2:** Pooled prevalence in people with serious mental illness

	**HIV**		**Hepatitis B virus**		**Hepatitis C virus**	
	Studies (n)	Prevalence (95% CI)	Studies (n)	Prevalence (95% CI)	Studies (n)	Prevalence (95% CI)
North America	21	6·0% (4·3–8·3)	2	2·2% (0·5–9·9)	13	17·4% (13·2–22·6)
Europe	5	1·9% (0·8–4·8)	4	2·7% (1·8–3·9)	6	4·9% (3·0–7·9)
Oceania	0	..	0	..	1	3·1% (1·0–9·3)
Africa	8	19·2% (14·4–25·2)	0	..	0	..
Asia	5	1·5% (1·0–2·4)	10	9·7% (0·6–15·3)	7	4·4% (2·8–6·9)
Central and South America	5	2·7% (0·8–8·2)	3	2·6% (1·0–6·1)	2	3·0% (1·8–5·0)

**Table 3:** Included studies of hepatitis B

	**Date**	**Location**	**Number of patients**	**HbAg (%)**	**Quality score**
**North America**					
Tabibian et al^63^	2008	USA	129	0·78	1
Wise et al^64^	2012	USA	115	4	2
**Europe**					
Gmelin et al^65^	1982	Germany	714	2·38	1
Porta et al^36^	1990	Spain	139	2·3	0
Di Nardo et al^66^	1995	Italy	206	4·8	1
Kakisi et al^40^	2009	Greece	805	2	1
**Asia**					
Tey et al^67^	1987	Singapore	71	12·7	1
Chaudhury et al^68^	1993	India	60	10	1
Chang et al^61^	1993	Taiwan	780	18·1	1
Chaudhury et al^69^	1994	India	100	11	1
Kimhi et al^70^	1997	Israel	121	32	0
Said et al^71^	2001	Jordan	192	7·29	1
Kuloglu et al^60^	2006	Turkey	255	10·1	3
Carey et al^53^	2007	India	948	3	4
Mamani et al^72^	2009	Iran	170	1·2	1
Hung et al^12^	2012	Taiwan	588	10·4	3
**Central and South America**					
de Souza et al^73^	2003	Brazil	433	1·6	3
Alvarado Esquivel et al^59^	2005	Mexico	99	7·1	3
Guimarães et al^57^	2009	Brazil	2238	1·6	3

**Table 4:** Included studies of hepatitis C virus in people with serious mental illness

	**Year**	**Location**	**Number of patients**	**Hepatitis C virus (%)**	**Quality score**
**North America**					
Al Jurdi et al^74^	2003	USA	238	16	3
Klinkenberg et al^31^	2003	USA	204	30	2
Osher et al^75^	2003	USA	668	18	3
Dinwiddie et al^76^	2003	USA	1556	8·5	3
Butterfield et al^77^	2003	USA	376	18·9	2
Rosenberg et al^32^	2005	USA	755	14	3
Freudenreich et al^78^	2007	USA	98	8·2	2
Tabibian et al^63^	2008	USA	129	38	1
Goldberg et al^79^	2008	USA	100	31	1
Matthews et al^80^	2008	USA	112	12	0
Rothbard et al^33^	2009	USA	588	21	2
Sockalingam et al^81^	2010	Canada	110	2·7	1
Himelhoch et al^35^	2011	USA	153	24·8	1
**Europe**					
Di Nardo et al^66^	1995	Italy	206	10·7	1
Cividini et al^82^	1997	Italy	1180	6·7	2
Stroffolini et al^83^	2003	Italy	526	5·1	1
Raja et al^84^	2006	Italy	1492	2·8	3
De Hert et al^39^	2009	Belgium	595	0·7	2
Kakisi et al^40^	2009	Greece	805	9	1
Central and South America					
Alvarado-Esquivel et al^56^	2008	Mexico	99	4·8	0
Guimarães et al^57^	2009	Brazil	2238	2·63	3
**Oceania**					
Gunewardene et al^85^	2010	Australia	95	3·1	1
**Asia**					
Chang et al^61^	1993	Taiwan	780	6·8	3
Kimhi et al^70^	1997	Israel	121	4·13	0
Sawayama et al^86^	2000	Japan	196	10·2	2
Nakamura et al^87^	2004	Japan	455	6·15	3
Kuloglu et al^60^	2006	Turkey	255	2·7	3
Mamani et al^72^	2009	Iran	170	1·8	1
Hung et al^12^	2012	Taiwan	588	1·9	3

**Table 5:** Sensitivity analysis

	**Adjustment**	**Odds ratio (95% CI)**	**p value**
HIV	None	1·00 (0·68–1·48)	0·99
HIV	Region	0·90 (0·67–1·21)	0·49
Hepatitis B	None	0·84 (0·46–1·55)	0·55
Hepatitis B	Region	0·69 (0·44–1·10)	0·11
Hepatitis C	None	0·92 (0·61–1·40)	0·69
Hepatitis C	Region	0·86 (0·63–1·19)	0·36

## References

[1] Bhugra D (2005). The global prevalence of schizophrenia. PLoS Med.

[2] BMA (2014). Recognising the importance of physical health in mental health and intellectual disability.

[3] Lagios K, Deane FP (2007). Severe mental illness is a new risk marker for blood-borne viruses and sexually transmitted infections. Aust N Z J Public Health.

[4] Cournos F, McKinnon K, Sullivan G (2005). Schizophrenia and comorbid human immunodeficiency virus or hepatitis C virus. J Clin Psychiatry.

[5] Campos LN, Guimaraes MD, Carmo RA (2008). HIV, syphilis, and hepatitis B and C prevalence among patients with mental illness: a review of the literature. Cad Saude Publica.

[6] Cournos F, McKinnon K (1997). Substance use and HIV risk among people with severe mental illness. NIDA Res Monogr.

[7] Public Health England. United Kingdom national HIV surveillance data tables. London, 2014.

[8] Health and Safety Executive Hepatitis B virus. http://www.hse.gov.uk/biosafety/blood-borne-viruses/hepatitis-b.htm.

[9] Public Health England. Hepatitis B epidemiology in London. London, 2012.

[10] Public Health England. Hepatitis C in the UK. London, 2014.

[11] Centers for Disease Control and Prevention Hepatitis B FAQs for health professionals. http://www.cdc.gov/hepatitis/hbv/hbvfaq.htm.

[12] Hung CC, Loh el W, Hu TM (2012). Prevalence of hepatitis B and hepatitis C in patients with chronic schizophrenia living in institutions. J Chin Med Assoc.

[13] Carey MP, Carey KB, Kalichman SC (1997). Risk for human immunodeficiency virus (HIV) infection among persons with severe mental illnesses. Clin Psychol Rev.

[14] Wong WCW, Cheung CSK, Hart GJ (2008). Development of a quality assessment tool for systematic reviews of observational studies (QATSO) of HIV prevalence in men having sex with men and associated risk behaviours. Emerg Themes Epidemiol.

[15] Clair WK, Eleazer GP, Hazlett LJ, Morales BA, Sercy JM, Woodbury LV (1989). Seroprevalence of human immunodeficiency virus in mental health patients. J S C Med Assoc.

[16] Hatem DS, Hurowitz JC, Greene HL, Sullivan JL (1990). Seroprevalence of human immunodeficiency virus in a state psychiatric institution. Arch Intern Med.

[17] Cournos F, Empfield M, Horwath E (1991). HIV seroprevalence among patients admitted to two psychiatric hospitals. Am J Psychiatry.

[18] Volavka J, Convit A, Czobor P, Douyon R, O'Donnell J, Ventura F (1991). HIV seroprevalence and risk behaviors in psychiatric inpatients. Psychiatry Res.

[19] Lee HK, Travin S, Bluestone H (1992). HIV-1 in inpatients. Hosp Community Psychiatry.

[20] Sacks M, Dermatis H, Looser-Ott S, Burton W, Perry S (1992). Undetected HIV infection among acutely ill psychiatric inpatients. Am J Psychiatry.

[21] Empfield M, Cournos F, Meyer I (1993). HIV seroprevalence among homeless patients admitted to a psychiatric inpatient unit. Am J Psychiatry.

[22] Meyer I, McKinnon K, Cournos F (1993). HIV seroprevalence among long-stay patients in a state psychiatric hospital. Hosp Community Psychiatry.

[23] Meyer I, Cournos F, Empfield M (1993). HIV seroprevalence and clinical characteristics of severe inpatient mentally ill homeless. J Soc Distress Homeless.

[24] Susser E, Valencia E, Conover S (1993). Prevalence of HIV infection among psychiatric patients in a New York City men's shelter. Am J Public Health.

[25] Stewart DL, Zuckerman CJ, Ingle JM (1994). HIV seroprevalence in a chronically mentally ill population. J Natl Med Assoc.

[26] Cournos F, Horwath E, Guido J (1994). HIV-1 infection at two public psychiatric hospitals in New York City. AIDS Care.

[27] Silberstein C, Galanter M, Marmor M, Lifshutz H, Krasinski K, Franco H (1994). HIV-1 among inner city dually diagnosed inpatients. Am J Drug Alcohol Abuse.

[28] Schwartz-Watts D, Montgomery LD, Morgan DW (1995). Seroprevalence of human immunodeficiency virus among inpatient pretrial detainees. Bull Am Acad Psychiatry Law.

[29] Doyle ME, Labbate LA (1997). Incidence of HIV infection among patients with new-onset psychosis. Psychiatr Serv.

[30] Krakow DS, Galanter M, Dermatis H, Westreich LM (1998). HIV risk factors in dually diagnosed patients. Am J Addict.

[31] Klinkenberg W, Caslyn RJ, Morse GA (2003). Prevalence of human immunodeficiency virus, hepatitis B, and hepatitis C among homeless persons with co-occurring severe mental illness and substance use disorders. Compr Psychiatry.

[32] Rosenberg SD, Drake RE, Brunette MF, Wolford GL, Marsh BJ (2005). Hepatitis C virus and HIV co-infection in people with severe mental illness and substance use disorders. AIDS.

[33] Rothbard AB, Blank MB, Staab JP (2009). Previously undetected metabolic syndromes and infectious diseases among psychiatric inpatients. Psychiatr Serv.

[34] Jackson-Malik P, McLaughlin MJ, O'Hara KT, Buxbaum LU (2011). Rapid oral fluid testing for HIV in veterans with mental health diagnoses and residing in community-assisted living facilities. J Assoc Nurses AIDS Care.

[35] Himelhoch S, Goldberg R, Calmes C (2011). Screening for and prevalence of HIV and hepatitis C among an outpatient urban sample of people with serious mental illness and co-occurring substance abuse. J Community Psychol.

[36] Porta M, Herrera R, Prats F, Yazbeck H, Gasso JM (1990). Absence of antibodies to HIV in short-, mid- and long-term institutionalized psychiatric patients in Barcelona. Eur J Epidemiol.

[37] Naber D, Pajonk F, Perro C, Lohmer B (1994). Human immunodeficiency virus antibody test and seroprevalence in psychiatric patients. Acta Psychiatr Scand.

[38] Ayuso-Mateos JL, Montanes F, Lastra I, Picazo de la Garza J, Ayuso-Gutierrez JL (1997). HIV infection in psychiatric patients: an unlinked anonymous study. Br J Psychiatry.

[39] De Hert M, Franic T, Vidovic D (2009). Prevalence of HIV and hepatitis C infection among patients with schizophrenia. Schizophr Res.

[40] Kakisi OK, Grammatikos AA, Karageorgopoulos DE, Athanasoulia AP, Papadopoulou AV, Falagas ME (2009). Prevalence of hepatitis B, hepatitis C, and HIV infections among patients in a psychiatric hospital in Greece. Psychiatr Serv.

[41] Acuda SW, Sebit MB (1996). Serostatus surveillance testing of HIV-I infection among Zimbabwean psychiatric inpatients, in Zimbabwe. Cent Afr J Med.

[42] Mashaphu S, Mkize DL (2007). HIV seropositivity in patients with first-episode psychosis. J Child Adolesc Ment Health.

[43] Collins PY, Berkman A, Mestry K, Pillai A (2009). HIV prevalence among men and women admitted to a South African public psychiatric hospital. AIDS Care.

[44] Singh D, Berkman A, Bresnahan M (2009). Seroprevalence and HIV-associated factors among adults with severe mental illness - a vulnerable population. S Afr Med J.

[45] Omoregie R, Efam MO, Ihongbe JC, Ogefere HO, Omokaro EU (2009). Seroprevalence of HIV infection among psychiatric patients in Benin City, Nigeria. Neurosciences.

[46] Henning MP, Kruger C, Fletcher L (2012). HIV sero-positivity in recently admitted and long-term psychiatric in-patients: prevalence and diagnostic profile. Afr J Psychiatry.

[47] Maling S, Todd J, van der Paal L, Grosskurth H, Kinyanda E (2011). HIV-1 seroprevalence and risk factors for HIV infection among first-time psychiatric admissions in Uganda. AIDS Care.

[48] Lundberg P, Nakasujja N, Musisi S, Thorson AE, Cantor-Graae E, Allebeck P (2013). HIV prevalence in persons with severe mental illness in Uganda: a cross-sectional hospital-based study. Int J Ment Health Syst.

[49] Dasananjali T (1994). The prevalence of HIV infection among mentally ill offenders in Thailand. J Med Assoc Thai.

[50] Chandra PS, Carey MP, Carey KB, Prasada Rao PSDV, Jairam KR, Thomas T (2003). HIV risk behaviour among psychiatric inpatients: results from a hospital-wide screening study in southern India. Int J STD AIDS.

[51] Tharyan P, Ramalingam S, Kannangai R, Sridharan G, Muliyil J, Tharyan A (2003). Prevalence of HIV infection in psychiatric patients attending a general hospital in Tamil Nadu, south India. AIDS Care.

[52] Chen C-H (1994). Seroprevalence of human immunodeficiency virus infection among Chinese psychiatric patients in Taiwan. Acta Psychiatr Scand.

[53] Carey MP, Ravi V, Chandra PS, Desai A, Neal DJ (2007). Prevalence of HIV, hepatitis B, syphilis, and chlamydia among adults seeking treatment for a mental disorder in southern India. AIDS Behav.

[54] Rodgers-Johnson PE, Hickling FW, Irons A (1996). Retroviruses and schizophrenia in Jamaica. J Mol Neurosci.

[55] Hutchinson GA, Simeon DT (1999). HIV infection rates and associated factors in high risk patients admitted to a psychiatric hospital in Trinidad and Tobago. West Indian Med J.

[56] Alvarado-Esquivel C, Arreola-Valenzuela MA, Rodriguez-Briones A (2008). Seroprevalence of selected viral, bacterial and parasitic infections among inpatients of a public psychiatric hospital of Mexico. Rev Inst Med Trop Sao Paulo.

[57] Guimarães MDC, Campos LN, Melo APS (2009). Prevalence of HIV, syphilis, hepatitis B and C among adults with mental illness: a multicenter study in Brazil. Rev Bras Psiquiatr.

[58] Gibson RC, Jackson S, Abel WD (2010). HIV seroprevalence among hospital inpatients with neuropsychiatric and other central nervous system disorders. West Indian Med J.

[59] Alvarado Esquivel C, Arreola Valenzuela MA, Mercado Suarez MF, Espinoza Andrade F (2005). Hepatitis B virus infection among inpatients of a psychiatric hospital of Mexico. Clin Pract Epidemol Ment Health.

[60] Kuloglu M, Gecici O, Atmaca M (2006). Hepatitis B and hepatitis C virus infection in institutionalized schizophrenia and other psychotic disorders patients in eastern Turkey. Neurol Psychiatry Brain Res.

[61] Chang TT, Lin H, Yen YS, Wu HL (1993). Hepatitis B and hepatitis C among institutionalized psychiatric patients in Taiwan. J Med Virol.

[62] Rosenberg SD, Goodman LA, Osher FC (2001). Prevalence of HIV, hepatitis B, and hepatitis C in people with severe mental illness. Am J Public Health.

[63] Tabibian JH, Wirshing DA, Pierre JM (2008). Hepatitis B and C among veterans on a psychiatric ward. Dig Dis Sci.

[64] Wise ME, Marquez P, Sharapov U (2012). Outbreak of acute hepatitis B virus infections associated with podiatric care at a psychiatric long-term care facility. Am J Infect Control.

[65] Gmelin K, Von Ehrlich-Treuenstatt B, Doerr HW (1982). Hepatitis A and B markers and presumable non-A, non-B hepatitis in a psychiatric institution. Zentralbl Bakteriol Mikrobiol Hyg A.

[66] Di Nardo V, Petrosillo N, Ippolito G (1995). Prevalence and incidence of hepatitis B virus, hepatitis C virus and human immunodeficiency virus among personnel and patients of a psychiatric hospital. Eur J Epidemiol.

[67] Tey BH, Oon CJ, Kua EH, Kueh YK, Wong YW, Chin JH (1987). Prevalence of hepatitis B markers in psychiatric in-patients in Singapore: a pilot study. Ann Acad Med Singapore.

[68] Chaudhury S, Chandra S, Chopra GS, Augustine M (1993). Australia antigen (HbsAg) in institutionalised schizophrenics. Indian J Psychiatry.

[69] Chaudhury S, Chandra S, Augustine M (1994). Prevalence of Australia antigen (HBsAg) in institutionalised patients with psychosis. Br J Psychiatry.

[70] Kimhi R, Barak Y, Achiron A, Elizur A (1997). Hepatitis among psychiatric inpatients: a high- risk group?. Int J Risk Saf Med.

[71] Said WM, Saleh R, Jumaian N (2001). Prevalence of hepatitis B virus among chronic schizophrenia patients. East Mediterr Health J.

[72] Mamani M, Hashemi SH, Niayesh A, Ghaleiha A, Hajilooei M (2009). Study on the frequency of hepatitis B and C infection in chronic psychiatric patients in Hamedan in 2006–2007. J Pak Med Assoc.

[73] de Souza MM, Barbosa MA, Borges AMT, Daher RR, Martins RMB, de Paula Cardosob DdD (2004). Seroprevalence of hepatitis B virus infection in patients with mental problems. Rev Bras Psiquiatr.

[74] Al Jurdi RK, Burress JW (2003). Prevalence of hepatitis C in psychiatric institutions. Psychosomatics.

[75] Osher FC, Goldberg RW, Goodman LA, Rosenberg SD (2003). Hepatitis C and individuals with serious mental illnesses. Psychiatr Ann.

[76] Dinwiddie SH, Shicker L, Newman T (2003). Prevalence of hepatitis C among psychiatric patients in the public sector. Am J Psychiatry.

[77] Butterfield MI, Bosworth HB, Meador KG (2003). Gender differences in hepatitis C infection and risks among persons with severe mental illness. Psychiatr Serv.

[78] Freudenreich O, Gandhi RT, Walsh JP, Henderson DC, Goff DC (2007). Hepatitis C in schizophrenia: screening experience in a community-dwelling clozapine cohort. Psychosomatics.

[79] Goldberg RW, Seth P (2008). Hepatitis C services and individuals with serious mental illness. Community Ment Health J.

[80] Matthews AM, Hauser P (2008). Hepatitis C screening in bipolar veterans. Addict Disord Their Treat.

[81] Sockalingam S, Shammi C, Powell V, Barker L, Remington G (2010). Determining rates of hepatitis C in a clozapine treated cohort. Schizophr Res.

[82] Cividini A, Pistorio A, Regazzetti A (1997). Hepatitis C virus infection among institutionalised psychiatric patients: a regression analysis of indicators of risk. J Hepatol.

[83] Stroffolini T, Marchi L, Brunetti E, Filice C (2003). Lack of hepatitis C transmission among institutionalized psychiatric patients. J Hepatol.

[84] Raja M, Azzoni A, Pucci D (2006). Characteristics of HCV positive patients in an Italian urban psychiatric unit. Clin Pract Epidemol Ment Health.

[85] Gunewardene R, Lampe L, Ilchef R (2010). Prevalence of hepatitis C in two inpatient psychiatry populations. Australas Psychiatry.

[86] Sawayama Y, Hayashi J, Kakuda K (2000). Hepatitis C virus infection in institutionalized psychiatric patients: possible role of transmission by razor sharing. Dig Dis Sci.

[87] Nakamura Y, Koh M, Miyoshi E (2004). High prevalence of the hepatitis C virus infection among the inpatients of schizophrenia and psychoactive substance abuse in Japan. Prog Neuropsychopharmacol Biol Psychiatry.

[88] Centers for Disease Control and Prevention (2015). Hepatitis C FAQs for Health Professionals. http://www.cdc.gov/hepatitis/hcv/hcvfaq.htm#a5.

[89] Carey MP, Ravi V, Chandra PS, Desai A, Neal DJ (2006). Screening for sexually transmitted infections at a DeAddictions service in south India. Drug Alcohol Depend.

[90] UNAIDS. UNAIDS Report on the global AIDS epidemic 2012. Joint United Nations Programme on HIV AIDS, 2012.

[91] Howard LM, Trevillion K, Khalifeh H, Woodall A, Agnew-Davies R, Feder G (2010). Domestic violence and severe psychiatric disorders: prevalence and interventions. Psychol Med.

[92] Lewis M, Allen H, Warr J (2010). The development and implementation of a nurse-led hepatitis C protocol for people with serious mental health problems. J Psychiatr Ment Health Nurs.

[93] Sanger C, Hayward J, Patel G (2013). Acceptability and necessity of HIV and other blood-borne virus testing in a psychiatric setting. Br J Psychiatry.

[94] Hughes E, Gray R (2009). HIV prevention for people with serious mental illness: a survey of mental health workers' attitudes, knowledge and practice. J Clin Nurs.

[95] Quinn C, Happell B, Browne G (2011). Talking or avoiding? Mental health nurses' views about discussing sexual health with consumers. Int J Ment Health Nurs.

[96] Aghaizu A, Brown A, Nardone A, Gill ON, Delpech VC (2013). HIV in the United Kingdom: 2013 report to end 2012.

[97] Costella ADG, Harris H, Hutchinson S (2013). Hepatitis C in the UK 2013 report.

[98] Weaver T, Madden P, Charles V (2003). Comorbidity of substance misuse and mental illness in community mental health and substance misuse services. Br J Psychiatry.

[99] Phillips P, Johnson S (2003). Drug and alcohol misuse among in-patients with psychotic illnesses in three inner-London psychiatric units. Psychiatr Bull.

[100] Carey MP, Carey KB, Maisto SA, Gordon CM, Vanable PA (2001). Prevalence and correlates of sexual activity and HIV-related risk behavior among psychiatric outpatients. J Consult Clin Psychol.

[101] McCann E (2010). The sexual and relationship needs of people who experience psychosis: quantitative findings of a UK study. J Psychiatr Ment Health Nurs.

[102] Lagios K, Deane F (2011). Psychiatrists' knowledge and practices in screening and assessment of hepatitis C for inpatients with severe mental illness. Australas Psychiatry.

