# Corrections

**DOI:** 10.1016/S2215-0366(15)00556-8

**Published:** 2016-01

*Hughes E, Bassi S, Gilbody S, Bland M, Martin F. Prevalence of HIV, hepatitis B, and hepatitis C in people with severe mental illness: a systematic review and meta-analysis.* Lancet Psychiatry *2016; **3:** 40–48*—In the first paragraph of the results section in this Article, the last sentence should have been: “With the addition of two papers from an updated search, we had 91 articles for quality assessment and meta-analysis”. In the fourth paragraph of the results, the statement that: “This set of participants was the largest of the three viral infections we investigated” was not true and has been removed. Also, the prevalence of serious mental illness was 17·4% in people with hepatitis C virus infection based on pooled data from 13 studies in North America. These corrections have been made to the printed Article, and to the online version as of January 6,2016.

